# TOMMY Trial (a comparison of tomosynthesis with digital mammography in the UK NHS breast screening programme) setting up a multicentre imaging trial

**DOI:** 10.1186/bcr2980

**Published:** 2011-11-04

**Authors:** FJ Gilbert, MGC Gillan, MJ Michell, KC Young, HM Dobson, J Cooke, H Purushothaman, YY Lim, SM Astley, SW Duffy

**Affiliations:** 1 University of Aberdeen, UK; 2 King's College Hospital, London, UK; 3 NCCPM, Guildford, UK; 4 West of Scotland Breast Screening Service, Glasgow, UK; 5 Jarvis Breast Screening Centre, Guildford, UK; 6 Barts & The London NHS Trust, London, UK; 7 University Hospital of South Manchester, Manchester, UK; 8 University of Manchester, UK; 9 Barts and The London School of Medicine and Dentistry, London, UK

## Introduction

Digital breast tomosynthesis (DBT) has the potential to improve the accuracy of standard digital mammography (DM) [1]. The TOMMY Trial is a multicentre, multireader, retrospective matched comparison of the diagnostic performance of DBT and DM.

## Methods

### Study population

Women (47 to 73 years old) recalled for further assessment after routine breast screening and women <50 years with a family history of breast cancer, attending annual mammographic screening. *Intervention *Women who consent to participate in the trial undergo standard two-view DM and DBT imaging of both breasts. Images are acquired in a single examination under the same degree of breast compression on a commercially available (Hologic) digital mammography system.

### Outcome measures

The primary outcome measure is the relative sensitivity and specificity of DM and DBT in the detection of early-stage cancers and subtle lesions, particularly in women with dense breasts. This will be evaluated in a retrospective reading study where readers at each centre conduct blinded independent reviews of anonymised DM, or DBT, or DM and DBT images of cases from other centres.

## Results

The trial set-up has involved lengthy and complex legal negotiation with collaborating sites, the equipment manufacturer and the grant-awarding body. Designated readers from each centre have completed tomosynthesis training and recruitment has commenced.

## Conclusion

It should be noted that the negotiation of contracts and commercial agreements adds a considerable time burden to the set-up phase of multicentre trials.

## References

[B1] Dobbins JT Godfrey DJ Digital X-ray tomosynthesis: current state of the art and clinical potential Phys Med Biol 2003 48 R65 R106 10.1088/0031-9155/48/19/R01 14579853

